# Domestic Medicine

**Published:** 1872-10

**Authors:** 


### Domestic Medicine.

### INFORMATION FOR THE HOUSEHOLD.

PREPARED BY THE EDITOR.

TO PRESERVE CLOTHES PINS.—They should be boiled a few moments and quickly dried, once or twice a month, when they become more flexible and durable. Clothes lines will last longer and keep in better order for wash-day service, if occasionally treated in the same way.

---

TOMATO CUSTARD.—This is said to be a beneficial diet for consumptives. It is made by straining finely stewed tomatoes through a coarse sieve, and adding two pints of milk and one pint of tomatoes, for four eggs and one teaspoonful of sugar. Bake in small cups quickly.

---

TO KEEP FRUIT.—Beat well together equal measures of honey and spring water in an earthen vessel; put in your peaches, plums and apricots, freshly gathered; cover closely, and they will keep fresh for a year. When taken out for use, they must be rinsed in cold water.

---

BROMIDE OF POTASSIUM FOR CONGESTIVE HEADACHES.—Dr. J. J. Brinkerhoff, Jr., of Auburn, N. Y., has always succeeded in preventing or in curing the so-called "sick headache," by fifteen-grain doses of bromide of potassium as often as may be required.

---

CASTOR OIL MADE PALATABLE.—The *Boston Journal of Chemistry* says that castor oil may be rendered as "sweet as honey" to take by combining it with an equal amount of glycerine, in which a few drops of oil of cinnamon has been rubbed up.

---

HOW TO CLEAN ALABASTER.—Take ground pumice stone of the finest quality, and mix it up with verjuice; let it stand for two hours, then dip in a sponge and rub the alabaster therewith; wash it with a linen cloth and fresh water, and dry it with clean linen rags. Any kind of marble may be done in the same manner.

---

TO KEEP TOMATOES WITHOUT CANNING.—The following is an approved recipe for keeping tomatoes the year round, so that they can hardly be distinguished from those fresh picked from the vines: Dissolve a teacupful of salt in a gallon of water. Pick ripe tomatoes, but not over-ripe, leaving a little of the stem on. They must be kept well covered with the brine.

---

A GOOD WHITEWASH.—Mix up half a pailful of lime and water. Then make a starch of half a pint of wheat flour, and pour it into the whitewash while hot. Stir it well, and it is ready for use. This is one of the simplest and best recipes, and the whitewash made by it will not rub off.

---

CHEAP SEALING WAX.—The following recipe furnishes a cheap sealing wax, useful for many purposes: Melt together two pounds of common beeswax, six ounces of turpentine, and two ounces of olive oil; add six ounces of red lead, boil a little, and stir until it is almost cold, then cast it into cold water, and make it up into rolls or cakes.

---

DETERSIVE PASTE FOR REMOVING GREASE FROM SILK.—Rub together fine French chalk and lavendar to the consistency of a thin paste, and apply thoroughly to the spots with the fingers; place a sheet of brown or blotting paper above and below the silk, and smooth it with a moderately heated iron. The French chalk may then be removed by brushing.

---

GLYCERINE CREAM.—This recipe is excellent:

Take of

Spermaceti, four drachms.
White wax, one drachm.
Oil of almonds, two troy-ounces.
Glycerine, one troy-ounce.

Melt the spermaceti, wax, and oil together, and when cooling stir in the glycerine and perfume.

---

CARBOLIC ACID IN SCARLET FEVER.—Dr. Cleaver (*Iowa Medical Journal*) has used the following formula with decided advantage in this disease:

Carbolic acid, 2 ounces.
Alcohol dilute, 2 ounces. Mix.

Mix a teaspoonful of this to a tablespoonful of water, used either as a gargle or with a mop, depending upon age and ability to gargle—say once every two hours. Also give ten to twenty drops of same in mucilage at same intervals—dose depending upon age of patient. This treatment was employed in 70 cases, with the most marked success.

TO PREVENT THE RUSTING OF STEEL INSTRUMENTS.—For this purpose the *Lancet* confidently recommends a mixture of equal parts of carbolic acid and olive oil, smeared over the surface of the instruments. This plan is much used by medical officers in the navy, and is found to preserve the polish and brightness of the steel, however moist and warm the climate may be.

---

TO CLEAN FLOOR CLOTHS.—Sweep and clean the floor cloths with a broom and damp flannel, in the usual manner, then wet them all over with milk, and rub them till bright with a dry cloth. They will thus look as well as if they were rubbed with a waxed flannel, without being slippery, or so soon clogged with dust or dirt.

---

TO CLEAN SILVER OR GOLD LACE.—Lay the lace smooth on a woolen carpet or piece of woolen cloth, and brush it free from dust, then burn rock alum and powder it fine, and afterwards sift it through a lawn sieve; then rub it over the lace with a fine brush, and in so doing it will take off the tarnish and restore it to its brightness. if it be not too much worn on the threads.

---

BOUQUET DE FOIN COUPE.—This perfume, or "New-mown Hay," as it is called, is prepared as follows:

| | |
|---|---|
| Extract of Tonka bean . . | 112 parts. |
| " geranium . . . | 56 " |
| " orange-flowers . . | 56 " |
| " rose . . . . | 56 " |
| " jasmine . . . | 56 " |
| " spirits of rose . . | 56 " |

Mix and filter.

---

TO CLEAN PAINT.—Never use a cloth, but take off the dust with a long haired brush.—With care, paint will look well for a length of time. When soiled, dip a sponge or a bit of flannel into soda and water, wash it off quickly, and dry immediately, or the strength of the soda will eat off the color.

When wainscot requires scouring, it should be done from the top downwards, and the soda be prevented from running on the unclean part as much as possible, or stains will appear after the whole is finished. One person should dry with old linen, whilst the other has scoured off the dirt and washed the soda off.

VARNISH FOR PHOTOGRAPHS.—The following German recipe affords a varnish for use on photographs, etc., which it is desired to re-touch, and give a surface which will take lead pencil well, and at the same time protect the photograph itself completely:

| | |
|---|---|
| Castor oil . . . . . | 1 part. |
| Sandarac . . . . . | 3 " |
| Alcohol . . . . . | 18 " |

Mix and dissolve.

---

TO KEEP POTATOES.—A western paper gives the following recipe for keeping potatoes, and asserts that it will preserve them for years:—Dust over the floor of the bin with lime; put in about six or seven inches of potatoes, and dust with lime as before. Put in six or seven inches of potatoes, and lime again, and repeat the operation till all are stowed away. One bushel of lime will do for forty bushels of potatoes, though more will not hurt them, the lime rather improving the flavor than otherwise. The lime may be used for fertilizing after this use of it.

---

FOR OFFENSIVE BREATH.—For removing this disagreeable affection, almost the only safe, effective thing to use, is the concentrated solution of chloride of soda; from six to ten drops of it in a wineglassful of pure spring water, taken immediately after the operations of the morning are completed. In some cases the odor arising from carious teeth is combined with that of the stomach. If the mouth be well rinsed with a teaspoonful of the solution of the chloride in a tumbler of water, the bad odor of the teeth will be removed.

---

RAILWAY AIR-CUSHIONS.—A writer to the *Medical Times* refers to the fatigue of the limbs produced after a long railway journey, as due mainly to the trembling motion of floor under the feet, and states that, having suffered considerably from this abuse, he was induced to try the experiment of using the well known air-cushion footstool. This answered so well that he has never traveled without using one in this way, and has found the effect to be a remarkable improvement.

---

TO CLEAN BRASS.—The *Technologist* says:—"Rub the surface of the metal with rotten stone and sweet oil, then rub off with a piece of canton flannel, and polish with soft leather. A solution of oxalic acid rubbed over tarnished brass soon removes the tarnish, rendering the metal bright. The acid must be washed off with water, and brass rubbed with whiting and soft leather. A mixture of muriatic acid and alum dissolved in water, imparts a golden color to brass articles that are steeped in it for a few seconds."

CURING OF HAMS AND BACON.—In curing hams and bacon it will be found advantageous to use the same quantity of common soda as of saltpetre—one ounce and a half of each to fourteen pounds of ham or a piece of bacon, using the usual quantity of salt. The soda prevents that hardness in the lean of the bacon which is so often found, and keeps it quite mellow all through, besides being a preventive of rancidity. This receipt has been very extensively tried for fifteen years, and invariably approved of.

POMATUM FOR CHAPPED LIPS.—The following is from the *Druggists' Circular*.—

℞

| | | |
|---|---|---|
| Lard | 16 | parts. |
| Cacao oil | 24 | " |
| Spermaceti | 8 | " |
| Yellow wax | 3 | " |
| Alcanna root | 1 | " |

The substances are fused for a quarter of an hour at a gentle heat, then strained through a cloth and mixed with—

| | | |
|---|---|---|
| Oil of lemon, | | |
| Oil of bergamot, of each | 1-6 | part. |
| Oil of bitter almonds | 1-15 | " |

when the mass is poured into suitable vessels to cool.

USE OF LEMONS.—When persons are feverish and thirsty beyond what is natural, indicated in some cases by a metalic taste in the mouth, especially after drinking water, or by a whitish appearance of the greater part of the surface of the tongue, one of the best "coolers" is to take a lemon, cut off the top, sprinkle over it some loaf sugar, working it down into the lemon with a spoon, and then suck it slowly, squeezing the lemon and adding more sugar as the acidity increases from being brought up from a lower point. Invalids with feverishness may take two or three lemons a day in this manner with the most marked benefit, manifested by a sense of coolness, comfort and invigoration. A lemon or two thus taken at "tea-time," is an entire substitute for the ordinary supper of summer, and would give many a man a comfortable night's sleep, and an appetite for breakfast, to which they are strangers, who will have their cup of tea, or supper of "relish" and "cake," and berries, and peaches and cream.

LOCAL APPLICATION TO BURNS.—Dr. Binikerd (*Med. and Surg. Reporter*) applies to burns, when seen early, carbolic acid and glycerine in the proportion of from 5 to 10 drops of the former, to 2 oz. of the latter. This is spread upon the burn by camel-hair pencil or feather, then a layer of white cotton retained by a roller bandage. For the suppurative stage, he employs the following:

Yellow wax melted and strained, 1 oz.
Raw linseed oil, 3 drachms.
Tannin, 1 drachm.
Subnitrate bismuth, grs. xx. Mix.

First melt the wax, add the oil, and stir the whole; then add the tannin, and lastly the bismuth. The ointment should be applied on pieces of lint.

